# Supplementary material for: Learning curve and surgical outcome of robotic assisted colorectal surgery with ERAS program
Source: Sci Rep. 2022 Nov 29;12:20566. doi: 10.1038/s41598-022-24665-w (PMC9709162; doi:10.1038/s41598-022-24665-w)
Supplement: Supplementary file 2 — Supplementary Information 2. [file 41598_2022_24665_MOESM2_ESM.zip › RAL Raw data-Ñ[▒K/115/hs_ñw╝╨░O▒Kñσ.pdf]

正式摘要(電子簽章：000000228397417)
W52 / W52 外文標題(注意:無英文姓名-請補病人基本檔!) 【讀取】憑證

# 臺中榮民總醫院

## 出院病歷摘要

轉入號： 轉出號：

[redacted]

| | |
|---|---|
| (8)流水編號 | |
| (9)入院日期 | 110/10/06 (2021) 直腸外科 W52 - 145 |
| (10)轉科日期 | |
| (11)出院日期 | 110/10/28 (2021) 住院天數計 22 日 |

### 診斷

- 入院 :Rectal cancer, cT3NxM0 s/p radiotherapy on 2021/9/30-10/6
- 出院 :Rectal cancer, cT3NxM0 s/p radiotherapy on 2021/9/30-10/6, s/p Robot assisted LAR+loop ileostomy on 2021/10/7, pT3N1a, with ostomy stenosis s/p revision on 2021/10/15

### 主訴

- bloody stool for about 1 month

### 病史

- This 63 years old male patient has past history of seizure for about 50 years under Neurology OPD follow up. He complained bloody stool for about 1 month. He came to LMD for help and rectum tumor was found. He was transferred to our OPD. Biopsy during sigmoid colon scope showed rectal adenocarcinoma. Suspected regional lymphadenopathy was found in CT. Thus, we started short course RT from 9/30 to 10/6. This time, he was admitted for surgical treatment.

### 體檢發現

- 
  - General appearance :
    - easy looking , conscious clear , without cardiopulmonary distress
    - Vital signs自動帶入date 體溫(BT) 脈搏(P) 呼吸(R) 血壓(BP) 身高(BH) 體重(BW) BMI
      20211006 35.8C 85/min 18/min 185/84mmHg 171.7cm 62.8Kg 21.3
  - HEENT :
    - Eyes: no anemic conjunctiva, no icteric sclera, normal EOM
    - ENT: no otorrhea, no rhinorrhea, no erythematous mucosa, no injected tonsil, no deformity
  - Neck: supple, freely movable, trachea midline,no deformity, no engorged jugular vein , no palpable thyroid or lymph node enlargement
  - Chest:
    - Inspection: Grossly normal, symmetric and fully expansion, no accessory muscle used, no operation scar, no spider angiomata, no gynecomastia
    - Palpation: no palpable mass or nodule, no subcutaneous emphysema, no tenderness
    - Ausculation: clear breathing sound
  - Heart:
    - Percussion: normal heart size
    - Ausculation: regular heart beat, normal S1, S2, no gallop, no murmur
  - Abdomen:
    - Inspection: in
      - flat contour, symetrically, no scar, no stria, no dilated veins, no rashes
    - Ausculation: normoactive bowel sound, no abdominal bruits
    - palpation: soft, no muscle guarding or rigidity, no local tenderness, no rebounding pain, no palpable mass, no palpable pulsation
    - Percussion: no shifting dulllness, no hepatomegaly, no splenomagaly
  - 

| Peripheral pulsation : | CA | BA | RA | FA | PA | PTA | DPA |
|---|---|---|---|---|---|---|---|
| Right side : | ++ | ++ | ++ | ++ | ++ | ++ | ++/+++ |
| Left side : | ++ | ++ | ++ | ++ | ++ | ++ | ++/+++ |

  - Genito-anus: no tenderness or palpable mass lesion or rectal shelf, normal external genitalia, normal anal tone, brown stool

**CRS CONDITION**

  - Colonoscope report: UP TO 10 CM FAV.RECTAL TUMOR AT 10 CM FAV WAS NOTED AND S/P BIOPSY.
  - CEA: 2.65 ng/ml
  - 
    - Lung

### 手術日期及方法

- 手術日期及方法20211007 1. Robotic (XI) assisted low anterior resection
  2. Intraoperative colonoscopy : (-).
  3. High ligation (+) : At IMA root (Via robotic clips). IMV was ligated via endoclip.
  4. Take down from the splenic flexure : (-)
  5. Robotic EndoGIA (45-4.3) X2 + EEA (29mm) + OpenGIA (80-3.8) X1 were applied to complete the side-to-end anastomosis
  6. Proximal cut end: > 10 cm, distal cut end: 5 cm
  7. No drainage tube was inserted
  8. ADHESION BARRIER FILM was placed
  9. Loop ileostomy (About 15~20cm from IC valve) was done over RLQ of abdomen
  20211015 Revision of colostomy or enterostomy complicated, deep

### 住院治療經過

- After admission, we removed Foley on POD2 and let him try diet under ERAS protocol. However, he complained abdominal fullness, pain on POD3. KUB showed dilated bowel loop. Thus, we let him NPO. Rectal tube was inserted and much ostomy output was found. NG tube was also inserted for decompression. We prescribed parenteral nutrition on POD5. Abdominal CT was done on 10/14 and dilated small intestine and stricture ostomy was found. Thus, operation with rivision of ileostomy was done on 10/15. NG tube was removed on 10/17. Much ostomy output was found after operation and abdominal cramping pain also improved. We let him try clear liquid diet and semiliquid diet then. Right lower abdomen firm mass without tenderness was found so we added Unasyn for empirical treatment. We declined IVF due to improved appetite. Under the stable condition, he was discharged on 10/28.

### 合併症

- ostomy stenosis s/p revision on 2021/10/15

### 一般檢查

- 累積報告 :

| DATE | TIME | ALB | TP | BIL.T | BIL.D | ALKP | AST | ALT | LDH | AMY | LIPASE | NH3 | Ferritin | UA |
|---|---|---|---|---|---|---|---|---|---|---|---|---|---|---|
| 1101007 | 1430 | | | 0.5 | | | | 15 | | | | | | |
| 1101011 | 1254 | 3.7 | 6.1 | 0.76 | 0.22 | 61 | 40 | 29 | 208 | | | | | |
| 1101012 | 1228 | | 6.2 | | | | | | | | | | | |
| 1101014 | 0852 | 3.4 | | 0.6 | 0.3 | | 18 | 27 | | | | | | |
| 1101017 | 1447 | 3.1 | | 0.5 | | | | 13 | | | | | | |
| 1101021 | 0902 | | | 0.49 | | | | 16 | | | | | | |
| 1101025 | 0849 | 3.5 | | 0.34 | | | | 34 | | | | | | |
| 1101028 | 0913 | 3.9 | | 0.39 | 0.14 | | 52 | 60 | | | | | | |

| DATE | TIME | WBC | RBC | HGB | HCT | MCV | PLT | BLST | NEUT | BAND | SEG | LYM | MON | EOS | BAS |
|---|---|---|---|---|---|---|---|---|---|---|---|---|---|---|---|
| 1101007 | 1431 | 7320 | | 12.5 | 34.8 | | 218 | | 93.7 | | | 3.3 | 2.9 | 0.0 | 0.1 |
| 1101011 | 1252 | 4300 | 4.33 | 13.3 | 39.8 | 91.9 | 199 | | 84.0 | | | 3.7 | 10.0 | 2.1 | 0.2 |
| 1101014 | 0911 | 4600 | 3.83 | 11.7 | 34.4 | 89.8 | 189 | | | 6.0 | 68.0 | 8.0 | 16.0 | 0.0 | 0.0 |
| 1101017 | 1444 | 5200 | | 11.0 | 32.7 | | 243 | | | | 81.0 | 5.0 | 13.0 | 0.0 | 0.0 |
| 1101021 | 0930 | 11780 | | 11.3 | 32.6 | | 363 | | | | 90.0 | 5.0 | 3.0 | 2.0 | 0.0 |

| | | | | | | | | | | | | | | | |
|---|---|---|---|---|---|---|---|---|---|---|---|---|---|---|---|
| 1101025 | 0907 | 5240 | | 11.8 | 35.3 | | 424 | | 79.2 | | | 8.4 | 8.6 | 3.4 | 0.4 |
| 1101028 | 0900 | 4710 | 3.98 | 12.0 | 36.3 | 91.2 | 413 | | 69.6 | | | 13.0 | 11.9 | 4.9 | 0.6 |

| DATE | TIME | NA | K | CL | BUN | CREAT | eGFR | CA | Mg | P | CRP | ESR | PCT | D-Dimer | LACTATE |
|---|---|---|---|---|---|---|---|---|---|---|---|---|---|---|---|
| 1101007 | 1430 | 133 | 3.8 | | 6 | 0.63 | 136.71 | | | | | | | | |
| 1101011 | 1254 | 132 | 3.9 | 94 | 13 | 0.79 | | 8.5 | | | | | | | |
| 1101012 | 1228 | | | | | | | | 2.3 | 2.5 | | | | | |
| 1101014 | 0852 | 132 | 3.2 | | 8 | 0.78 | 106.85 | 7.8 | | | | | | | |
| 1101017 | 1447 | 136 | 3.8 | | 10 | 0.68 | 125.18 | 7.9 | | | | | | | 13.8 |
| 1101021 | 0902 | 133 | 3.8 | | 8 | 0.83 | 99.46 | | | | | | | | |
| 1101025 | 0849 | 137 | 3.7 | | 3 | 0.74 | 113.54 | 8.1 | | | | | | | |
| 1101028 | 0913 | 139 | 4.4 | | 6 | 0.89 | 91.76 | | | | | | | | |

| DATE | TIME | GLUCOSE | HbA1C | CHOL | TG | HDL | LDL | KETONE | CK | CKMB | TRO-T | PT-P | APTT-P | NT-pro BNP |
|---|---|---|---|---|---|---|---|---|---|---|---|---|---|---|
| 1101012 | 1228 | | | 141 | 48 | 65 | 63 | | | | | | | |

- 放射線報告 :檢驗項目 **:ABDOMEN (KUB),SUPINE** 報告日期 **:1101022**

工 訴 :
Result:N
Finding:
> Gaseous distension of bowel loop, ileus or obstruction should be considered. Suggest follow-up.
> Bilateral psoas and renal shadows were partially obscured by bowel gas.
> S/P ostomy over RLQ abdomen
> Surgical suture over pelvic region
> Pelvic phleboliths
> Mild degenerative change of lumbar spine.
Impression:
As above description.

檢驗項目 **:CT ABDOMEN-SURVEY** 報告日期 **:1101018**
主 訴 :
Result:Y
Finding:
CT of abdomen with IV contrast:
1. History of rectal cancer, S/P R/T, LAR & loop ileostomy for F/U.
2. Slight wall thickening at rectal anastomotic site, R/O post-operative change firstly. Suggest follow up to exclude local recurrence.
3. Diffuse dilatation of small intestine without obvious transitional zone, ileus or partial obstruction could not be ruled out. Please correlate with clinical status.
4. Mild stranding of peritoneal & pelvic fat plane, R/O post-operative change firstly. Suggest F/U to exclude peritoneal carcinomatosis.
5. Mild stranding of subcutaneous fat plane at anterior abdominal wall and subcutaneous emphysema at anterior abdominal wall & inguinal area, R/O post-operative change
6. Small gallbladder stones
7. Liver, pancreas, both kidneys, spleen are without specific abnormality.
8. Post nasogastric tube placement
9. Mild increasing infiltration over LLL, R/O inflammatory process.
10.A tiny ground glass nodule about 2mm in size at RLL(ax.8)
Impression:
1. rectal cancer, S/P R/T, LAR & loop ileostomy with slight wall thickening at rectal anastomotic site, R/O post-operative change firstly. Suggest follow up to exclude local recurrence
2. Diffuse dilatation of small intestine without obvious transitional zone, ileus or partial obstruction could not be ruled out. Please correlate with clinical status.
3. Other findings, as above description

檢驗項目 **:ABDOMEN (KUB),SUPINE** 報告日期 **:1101015**
工 訴 :
Result:N
Finding:
> Diffuse dilated small bowel loops with paucity of gas in colon, suspect small bowel obstruction. Suggest follow up.
> Subcutaneous emphysema over right abdomen region
> S/P ostomy over RLQ abdomen
> Surgical suture over pelvic region
> Pelvic phleboliths
> Mild DJD change of T-L spine
Impression:
As above description.

特殊檢查

病理報告

- 活體組織切片病理報告　手術日期：**110/10/07**　病理號：**S1032083**

診斷醫師:陳律臻 報告日期:110/10/15
Pathologic diagnosis:
1. Rectum, low anterior resection --- Adenocarcinoma, moderately differentiated. Tumor invaded to perirectal soft tissue, and metastasized to perirectal lymph nodes (1/14). The bilateral cut ends are free of tumor.
2. Rectum, cut end, distal, resection --- Unremarkable.
3. IMA root, dissection --- Unremarkable lymph nodes (0/3).
Ancillary study for diagnosis:
IHC stains for PMS2 and MSH6 are done on section J. Both markers are preserved.
Prognostic and predictive factor:
1. Tumor size: 4.5 cm.
2. Depth of invasion: Perirectal soft tissue.
3. Histologic grade: G2: Moderately differentiated.
4. Angiolymphatic invasion: Not identified.
5. Perineural invasion: Not identified.
6. Tumor deposits (discontinuous extramural extension): Present, specify number of deposits: 2.
7. Macroscopic tumor perforation: Not identified.
8. Surgical Margins: a) Proximal Margin: Uninvolved by invasive carcinoma, distance of tumor from margin: 6.5 cm. b) Distal Margin: Uninvolved by invasive carcinoma, distance of tumor from margin: 2 cm. c) Radial or Mesenteric Margin: Uninvolved by invasive carcinoma, distance of tumor from margin: 1.5 cm.
9. Lymph node status: Involved (1/17).
10. Extranodal involvement: Present.
11. Tumor regression grading S/P CCRT: No known presurgical therapy.
12. Type of polyp in which invasive carcinoma arose: Not identified.
13. Additional pathologic findings: Not identified.
14. Pathological TNM Stage: pT3N1a(According to the eighth edition, American Joint Committee on Cancer Staging Guidelines for Tumors).
15. TNM descriptors: Not applicable.
Gross description:
The specimen consists of 1) a segment of rectum, 13 cm in length and 4 cm in proximal cut end circumference, 6 cm in distal circumference, and perirectal soft tissue. A gray tan firm annular elevated and centrally ulcerative tumor, 4.5x4.5 cm, is noted, 2 cm to the distal cut end. On cross section, the tumor invades to the perirectal soft tissue with 15 mm to the radial margin. The both cut ends are free of

- tumor grossly. 2) distal cut end, a piece of tan mucosa-covered soft tissue, 2x1.5x1.5 cm. 3) IMA root, a piece of tan yellow soft tissue, 3x2x1 cm. Representative part for section: A) specimen 2 B) specimen 3 C) proximal cut end D) distal cut end E-J) tumor K-N) lymph nodes.
  Note:
  1. The interpretation of MMR panel and recommended studies are as below: (1) No loss of nuclear expression of MMR proteins: low probability of microsatellite instability-high (MSI-H). (2) Loss of nuclear expression of MLH1 and PMS2: testing for methylation of the MLH1 promoter and/or mutation of BRAF is indicated (the presence of a BRAF V600E mutation and/or MLH1 methylation suggests that the tumor is sporadic and germline evaluation is probably not indicated; absence of both MLH1 methylation and of BRAF V600E mutation suggests the possibility of Lynch syndrome, and sequencing and/or large deletion/duplication testing of germline MLH1 may be indicated). (3) Loss of nuclear expression of MSH2 and MSH6: high probability of Lynch syndrome (sequencing and/or large deletion/duplication testing of germline MSH2 may be indicated, and, if negative, sequencing and/or large deletion/duplication testing of germline MSH6 may be indicated). (4) Loss of nuclear expression of MSH6 only: high probability of Lynch syndrome (sequencing and/or large deletion/duplication testing of germline MSH6 may be indicated). (5) Loss of nuclear expression of PMS2 only: high probability of Lynch syndrome (sequencing and/or large deletion/duplication testing of germline PMS2 may be indicated). (6) Please note: There are exceptions to the above IHC interpretations. These results should not be considered in isolation, and clinical correlation with genetic counseling is recommended to assess the need for germline testing.
  2. This case has been peer reviewed by two doctors.
  #T-59600_2 #M-81403_2 3 1105 S0000F
  #T-C4560_2 #M-81406_2 3 1105 000000

## 其它

## 出院時情況

- 3.改門診治療

## 出院指示

| 藥名 | 劑量 | 劑量單位 | 用法 | 頻次 | 數量 | 數量單位 | 現況 | 天數 | 單位 |
|---|---|---|---|---|---|---|---|---|---|
| Keppra tab 500mg | 2 | TAB | PO | BID | 52 | TAB | TH | 13 | 天 |
| $Biofermin tab | 2 | TAB | PO | TID | 78 | TAB | TH | 13 | 天 |
| Otilonium tab 40mg | 1 | TAB | PO | BID | 26 | TAB | TH | 13 | 天 |
| Frisium tab 10mg | 1 | TAB | PO | BID | 26 | TAB | TH | 13 | 天 |
| Tegretol CR-200mg | 2 | TAB | PO | BID | 52 | TAB | TH | 13 | 天 |
| Vesicare tab 5mg | 1 | TAB | PO | QD | 13 | TAB | TH | 13 | 天 |
| Econ tab 40mg | 1 | TAB | PO | QID | 52 | TAB | TH | 13 | 天 |
| MOSAD tab 5mg | 1 | TAB | PO | TID | 39 | TAB | TH | 13 | 天 |
| Tamokas PR tab 0.4mg | 1 | TAB | PO | QDAC | 13 | TAB | TH | 13 | 天 |
| Panho tab 40mg | 1 | TAB | PO | QDAC | 13 | TAB | TH | 13 | 天 |
| Unasyn TAB 375mg | 1 | TAB | PO | Q12H | 14 | TAB | TH | 7 | 天 |

## 出院計劃

主治醫師 2062F 蔣鋒帆 / 住院醫師 2331F 陳凱祥

**正式摘要(電子簽章：000000228397417)**

W52 / W52

doctorRank:B
